# A self-help mobile messaging intervention to improve subthreshold depressive symptoms among older adults in a socioeconomically deprived region of Brazil (PRODIGITAL): a pragmatic, two-arm randomised controlled trial

**DOI:** 10.1016/j.lana.2024.100897

**Published:** 2024-10-07

**Authors:** Carina Akemi Nakamura, Nadine Seward, Tim J. Peters, Thiago Vinicius Nadaleto Didone, Felipe Azevedo Moretti, Marcelo Oliveira da Costa, Caio Hudson Queiroz de Souza, Gabriel Macias de Oliveira, Monica Souza dos Santos, Luara Aragoni Pereira, Mariana Mendes de Sá Martins, Pepijn van de Ven, William Hollingworth, Ricardo Araya, Marcia Scazufca

**Affiliations:** a Departamento de Psiquiatria, Faculdade de Medicina FMUSP, Universidade de Sao Paulo, Sao Paulo, Brazil; b Department of Global Health and Social Medicine, Harvard Medical School, Boston, MA, USA; c Health Service and Population Research, Institute of Psychiatry, Psychology and Neuroscience, King's College London, London, UK; d Department of Clinical Psychology, Health and Social Science, University of Edinburgh, Edinburgh, UK; e Bristol Dental School, University of Bristol, Bristol, UK; f Instituto de Psiquiatria, Hospital das Clinicas HCFMUSP, Faculdade de Medicina, Universidade de Sao Paulo, Sao Paulo, Brazil; g Health Research Institute, University of Limerick, Limerick, Ireland; h Health Economics Bristol, Population Health Sciences, Bristol Medical School, University of Bristol, Bristol, UK

**Keywords:** Subthreshold depression, Depression, Digital intervention, Psychosocial intervention, Older adults, Primary healthcare, LMIC, Brazil, Randomised controlled trial

## Abstract

**Background:**

Subthreshold depression is a risk factor for major depression and is associated with increased morbidity and mortality, especially in older adults. There is emerging evidence that digital interventions, including self-help interventions, may reduce depressive symptoms. We aimed to evaluate the effectiveness of a mobile messaging intervention at reducing subthreshold depressive symptoms among older adults in Brazil.

**Methods:**

PRODIGITAL was a single blind, two-arm, individually randomised controlled trial conducted in 46 primary care clinics in the city of Guarulhos, Brazil. Individuals aged 60+ years were contacted by phone following a randomly ordered list for a screening assessment. Those who presented with anhedonia and/or depressed mood (Patient Health Questionnaire (PHQ)-2≥1), and who subsequently scored between 5 and 9 on the PHQ-9 were invited to participate. The intervention arm received the ‘Viva Vida’ digital self-help intervention consisting of automated multi-media messages sent via WhatsApp. Forty-eight audio and visual messages based on psychoeducation and behavioural activation were automatically delivered over six weeks. The control arm received a single message containing information about depression. The primary outcome was the difference in mean PHQ-9 scores between treatment arms at the three-month follow-up. All primary analyses were performed according to allocated arm with imputed data. The trial is registered with ReBEC, RBR-6c7ghfd.

**Findings:**

Participants were recruited between 8 September 2021 and 19 August 2022. Of the 454 participants enrolled, 223 were randomised to the intervention arm, 231 to the control arm. Participants’ mean age was 65.3 years (SD 5.0) and 64.0% (n = 292) were female. A total of 385 (84.8%) completed the three-month follow-up assessment; no difference in mean PHQ-9 scores between the treatment arms was observed (adjusted difference: −0.61; 95% CI: −1.75, 0.53; *p* = 0.29).

**Interpretation:**

These results demonstrate that the Viva Vida digital self-help intervention did not help to improve subthreshold depressive symptoms amongst older adults. Further research is needed to understand why this self-help intervention was not effective in this population, and to explore how it might be adapted to achieve this goal.

**Funding:**

10.13039/501100001807São Paulo Research Foundation and UK Joint Global Health Trials.

**Research in context**

**Evidence before this study**

We searched PubMed for randomised controlled trials on Dec 14, 2023, using the search terms (digital OR internet OR web OR mobile OR e-health OR technology OR automated OR self-help OR self-guided OR WhatsApp) AND (subthreshold OR subclinical OR subsyndromal) AND (depression OR depressive) AND (older OR elderly OR senior). We did not apply any restrictions on languages or date of publication. Two studies assessing digital interventions for subthreshold depression without any kind of help from health professionals or lay groups were found. An internet-based cognitive behavioural therapy (iCBT) was delivered to adults aged 50+ years in the Netherlands. In the other study conducted in Australia automated emails using self-help behaviour strategies (Mood Memos) were sent to adults from 18 to 78 years (mean age of 36 years). The interventions showed improvement in depressive symptoms at ten (iCBT) and six weeks (Mood Memos).

**Added value of this study**

We report a digital self-help psychosocial intervention that was not supported by any health professionals or lay groups, for subthreshold depressive symptoms among older adults in Brazil. To the best of our knowledge, this is the first psychosocial intervention of this kind in a low- and middle-income country. The intervention was designed to be user-friendly and accessible to an older population with low literacy and limited digital skills. While a substantial proportion of the participants opened the majority of the messages, indicating that they were at least interested in the programme, this does not necessarily indicate that the messages were read or listened to fully, let alone acted on, and in the event, we did not observe any difference between trial arms in reducing subthreshold depressive symptoms.

**Implications of all the available evidence**

Digital self-help interventions with no support of health professionals or lay groups are usually low cost and can easily be scaled up. Consequently, they have the potential to improve access to care and help reduce the mental health treatment gap in an affordable way. Future studies should focus on understanding how to adapt the Viva Vida self-help intervention to be effective for subthreshold depression and, consequently, prevent major depression.

## Introduction

Depression is widely recognised as a leading cause of disability globally.^1^ Although depression is common across ages, older individuals with depression have increased risks of disability, mortality, and poorer outcomes from physical health.^2^ Suggestions that depression plays a central role in multimorbidity in older populations highlights it as a potential target for interventions aimed at improving overall health outcomes.^3^

Subthreshold depression is commonly defined as having one core symptom of depression, such as anhedonia and depressed mood, along with mild depressive symptoms that do not meet the criteria for major depressive disorder.^4^^,^^5^ Research suggests that subthreshold depression is an important risk factor for major depression, with studies indicating that between 10% and 50% of people with subthreshold depression develop major depression.^4^ The prevalence of subthreshold depression in older adults is around 10% in community settings and 20% in primary care.^4^ In Brazil, a 14% prevalence of subthreshold depressive symptomatology was observed among older adults living in socioeconomically deprived areas.^6^ As with major depression, older adults with subthreshold depression are at greater risk of worsening symptoms of comorbidities and functional decline, increased risk of mortality, and increased healthcare costs.^7^ Thus, there is an urgent need to develop and evaluate scalable interventions to manage subthreshold depression, especially in Low- and Middle-Income Countries where the need is greatest.

The use of digital interventions to manage depressive symptoms is becoming increasingly popular,^8^ with growing evidence that they may help to improve symptoms of subthreshold and major depression.^9^ The COVID-19 pandemic has further spotlighted the necessity for alternatives to in-person care. However, studies in Low- and Middle-Income Countries testing digital interventions to improve symptoms of depression in older adults are limited, particularly those with no involvement of mental health professionals.^8^, ^9^, ^10^ Unguided, self-help digital interventions have been suggested as a treatment option for individuals with mild symptoms of depression in contrast to guided interventions.^9^ Since self-help interventions can be both low cost and easily scalable, they could potentially serve as an important strategy to reduce the mental health gap in Low- and Middle-Income Countries.^11^^,^^12^

We previously developed and evaluated a task-shared psychosocial intervention delivered by community health workers supported by a dedicated digital application (PROACTIVE) for depression among older adults.^13^, ^14^, ^15^ During the screening phase of the PROACTIVE study, we found that 46% of older adults reported using WhatsApp.^16^ Given the effectiveness of PROACTIVE,^15^ and capitalising on the high proportion of the population of all ages using WhatsApp in Brazil, we developed a digital self-help psychosocial intervention (Viva Vida) for the management of depressive symptoms. The PROACTIVE psychoeducation and behavioural activation contents were adapted to be automatically delivered via WhatsApp with no support of health professionals. The Viva Vida version for depression was evaluated in the PRODIGITAL-D randomised controlled trial that found a short-term effect on improving from depressive symptoms.^17^^,^^18^ Here we evaluate the effectiveness of the Viva Vida programme for the management of subthreshold depressive symptoms among older adults in Guarulhos, Brazil (PRODIGITAL).

## Methods

### Study design and participants

PRODIGITAL was a two-arm, single blind randomised controlled trial with a 1:1 allocation ratio of individuals. The study took place in Guarulhos city, located in the metropolitan region of São Paulo city with 1.3 million inhabitants (of which 13.8% is 60+ years). The Family Health Strategy is the main primary care model in Brazil and covers 31% of the Guarulhos population, mainly living in the most socioeconomically vulnerable areas. While this model aims to provide more comprehensive care through multi-professional teams with preventive and health promotion approaches, the traditional primary care model relies mostly on medical appointments to deliver primary care assistance. In this study, we included 46 primary care clinics, known as Unidade Básica de Saúde (UBS), of which 37 were based on the Family Health Strategy model and nine were a mix of both models. Only individuals receiving Family Health Strategy care were eligible.

Inclusion criteria were: registered at a participating UBS; age 60+ years; access to WhatsApp; and subthreshold depressive symptomatology. The latter criterion was assessed using the Patient Health Questionnaire (PHQ) and defined as at least one core symptom of depression^4^ (PHQ-2 score≥1), and mild depressive symptoms (PHQ-9^19^ scores between 5 and 9). Exclusion criteria included: significant vision or hearing impairment (not being able to listen or see messages received on the mobile phone); and significant cognitive impairment or language barriers (not being able to understand the instructions or questions during the assessment). Also excluded were participants of our previous randomised controlled trial (PROACTIVE),^15^ and household members of those already enrolled in the study. Participants who answered positively to the 9th item of the PHQ-9 (suicidal ideation) and were deemed as high suicidal risk by a standardised questionnaire were also excluded.

Verbal consent was granted for the screening assessment and for participation in the trial. This study was authorised by the Secretaria da Saúde do Município de Guarulhos and approved by the Ethics Committee of the Hospital das Clínicas da Faculdade de Medicina da Universidade de São Paulo—HCFMUSP (CAPPesq, ref: 4.144.603).

### Randomisation and masking

Participants were stratified by age (60–69, 70–79 years, 80+ years), sex (male/female), and type of UBS (full Family Health Strategy/mixed models). The randomisation sequence was generated using random permuted blocks with random block sizes of six, eight or ten by researchers not directly involved in the data collection (CAN and TJP). The ‘randomization module’ of the Research Electronic Data Capture (REDCap)^20^ was used to conceal the randomisation sequence and randomise participants in the randomised controlled trial.

Independent research assistants responsible for collecting data were masked to trial allocation, and two different teams were assigned for either the first or the second follow-up assessment. Also, whenever possible, research assistants did not perform more than one interview with the same participant. It was not possible to mask researchers responsible for scheduling the automated delivery of messages, but they had no role in collecting data. Trial participants were not masked due to the nature of the intervention.

### Procedures

Screening for eligibility was conducted simultaneously with the PRODIGITAL-D randomised controlled trial^17^^,^^18^ that recruited older adults with depressive symptomatology (PHQ-9 scores≥10). An alphabetical list of the names and phone numbers of all older individuals registered with the eligible UBSs and receiving care from the Family Health Strategy were provided by Guarulhos Health Secretary. Duplicate entries, individuals with no phone numbers, and PROACTIVE randomised controlled trial^15^ participants were excluded. The remaining individuals received a random identification number (due to the alphabetical ordering of potential participants) when entered into the REDCap system. Details of the recruitment process have been published.^21^

Using the randomly ordered list of identification numbers, a pre-screening phase checked if the mobile phone numbers were valid and registered with WhatsApp. Those with a valid WhatsApp number were contacted by phone for the screening assessment. Individuals were first screened for inclusion and exclusion criteria, including the PHQ-2.^22^ If anhedonia or depressed mood was present (PHQ-2 scores≥1), then the remaining seven questions of the PHQ-9 were asked. Those who scored between 5 and 9 on the PHQ-9 were considered to have subthreshold depressive symptomatology^6^ and completed a baseline questionnaire regarding other mental and physical health conditions, quality of life, and sociodemographic profile. The screening assessment and baseline questionnaire together took approximately 35 min.

Individuals who completed the baseline assessment were invited to participate, with research assistants providing all information about the trial (taking approximately 15 min). If the participants were not able to spend this extra time, this was completed in an additional call no more than 28 days after the PHQ-9 assessment. All assessments were conducted by phone and the calls were recorded if authorised by the individual.

Following consent, participants were randomised to either the intervention or control arm. A list with new participants for each treatment arm was sent at the end of each week to the team responsible for sending the Viva Vida messages. The first message (intervention arm) and the single message (control arm) were sent out no later than ten days after consenting to the randomised controlled trial. Participants in both arms continued to receive the usual care provided by the UBSs, and the research team did not interfere in any clinical decisions (medications, consultations, and treatments). Although UBS staff were not involved in the trial, participants in both arms were encouraged to seek healthcare if they felt this was needed.

Participants in the intervention arm received a version of the Viva Vida programme developed for older adults with subthreshold depressive symptoms. The programmes for depressive and subthreshold depressive symptoms have the same structure, but differ in the content of the messages (how the story is told), and the proportion of the audio messages (83% and 50% of total messages in the programme for depressive and subthreshold depressive symptoms, respectively). The contents are based on our effective PROACTIVE psychosocial intervention.^15^ Viva Vida for subthreshold depressive symptoms is a six-week digital psychosocial intervention based on psychoeducation and behavioural activation. The programme was specifically designed for older adults with low levels of education and consisted of 48 audio and visual messages automatically delivered by WhatsApp four days a week (twice a day). Audio messages using storytelling techniques with a duration of 3 min were delivered in the morning. The audio message introduced a topic that was reinforced by a visual message (summary diagrams) sent in the afternoon. Topics covered during the programme were about depressive symptoms and ways to deal with them, including adding more pleasant and/or meaningful activities in their daily lives. The messages can be initially accessed from anywhere with an internet connection and once downloaded to the participant's mobile phone, they can be accessed at any time and as often as desired. In addition to the 48 messages, once a week participants received a message with a question asking their opinion and/or experiences with the programme. Participants were able to answer the question using the WhatsApp ‘quick reply’ tool, in which up to three pre-defined responses were offered. They were also invited to send a text or audio message to expand on their answers. All spontaneous messages sent by participants were replied with an automated message. At the beginning of the programme, participants were informed that they should contact the technical support if they had issues receiving the messages.

Participants in the control arm received one audio message approximately 6 min long. The message addressed depressive symptoms, provided guidance on their management, and included tips for maintaining a healthier lifestyle.

A dedicated web application interfacing with WhatsApp using the WhatsApp Business Application Programming Interface was developed to schedule and deliver the messages. The system was hosted on a cloud service with all communication encrypted using Secure Socket Layer certificates. The web application collected and managed the WhatsApp data, including timestamps (date and time) indicating when messages were sent, delivered, and ‘opened’.

During the first two weeks of the programme, the research team monitored the status of the messages to identify and contact participants with technical issues (such as those who had not received or opened the messages). This enabled identification and correction of potential issues with the system, ensuring that participants received the messages as intended. After this period, no additional contact was made from the research team until the follow-up assessments.

Follow-ups were completed by phone at three months (weeks 12–16) and five months (weeks 20–24) after sending the first message. A four-week window was used to maximise response to follow-up considering the reality of conducting phone interviews. Information collected in the assessments included measures on depressive symptomatology (PHQ-9),^19^ anxiety symptomatology assessed using the Generalised Anxiety Disorder-7 (GAD-7),^23^ health-related quality of life with the European Quality of Life five-dimensional questionnaire, five-level version (EQ-5D-5L),^24^ capability wellbeing assessed with the ICEpop CAPability measure for Older people (ICECAP-O),^25^ and perceived loneliness assessed using the 3-item University of California, Los Angeles (UCLA) loneliness scale (3-item UCLA).^26^

All assessment data were collected and managed using REDCap^20^ hosted at the Hospital das Clínicas da Faculdade de Medicina da Universidade de São Paulo. Quality control of a random sample of recorded assessments was conducted by an independent research assistant (not involved in data collection) to ensure all procedures had been followed and the quality of data collected.

Severe adverse events, including acute suicidal risk, hospitalisations and death were recorded during the follow-up assessments for participants in both study arms. Participants who reported suicidal ideation were assessed for acute suicidal risk by a standardised protocol used in a previous study.^15^ UBS managers were contacted and informed about participants with suicidal ideation. Hospital admissions and deaths were investigated with participants and/or family members to understand if these were related to study participation. The causes of hospitalisation are reported in the Supplementary Table S1. The specific causes of death were not collected due to the sensitivity of the topic; family members were only asked to confirm if it was related to mental health issues.

### Outcomes

The primary outcome was depressive symptoms at three months, represented by the (mean) PHQ-9 score in each trial arm. This outcome was also assessed at five months as a secondary outcome. Additional secondary outcomes measured at both three and five months were: depressive symptomatology defined as PHQ-9 scores≥10,^19^ anxiety symptomatology (GAD-7),^23^ health-related quality of life (EQ-5D-5L),^24^ capability wellbeing (ICECAP-O),^25^ and loneliness (3-item UCLA).^26^

### Sample size

We aimed for a large and clinically significant difference in mean depressive symptoms between the intervention and control arms of 0.33 standard deviations in PHQ-9 scores.^27^ A sample size of 142–162 individuals in each arm (284–324 in total) can detect this difference at three months with 80%–85% power with a two-sided 5% significance level. Albeit for a different study group and intervention, our PROACTIVE pilot study indicated that this effect size is feasible with psychosocial interventions (PHQ-9 means of 5.9 and 6.4 with a standard deviation of 1.5; data not published). We anticipated 25% attrition and therefore aimed to recruit 225 individuals in each arm for a total sample size of 450.

### Statistical analysis

#### Primary and secondary outcomes

The primary and secondary outcomes of mean PHQ-9 scores, compared between the intervention and control arms at three and five months respectively, were evaluated using linear regression models that assume residuals are normally distributed. The secondary outcomes of (mean scores of) GAD-7, EQ-5D-5L, ICECAP-O, and 3-item UCLA at three and five months were also evaluated using linear regression models. The secondary outcome of risk of depressive symptomatology (defined as PHQ-9≥10), indicative of clinical depression,^19^ at three and five months was evaluated using fixed effects Poisson regression models with a log-link function and robust error variances. All regression analyses were adjusted for stratification (age group, sex, and type of UBS) and baseline values of the corresponding outcome.

Primary analyses were performed by intention to treat with imputed data. For each arm separately, missing data for all outcomes were replaced using multiple imputation by chained equations (MICE) as implemented in the MI command in Stata 17 under the assumption that data were missing at random (MAR). Supplementary Material provides details of the methods used for the missing data analyses including sensitivity analyses as well as results comparing estimates using complete case versus imputed data (Supplementary Materials, pages 2–9, Supplementary Tables S2–S8).

#### Subgroup analyses

Pre-specified subgroup analyses were investigated using the Wald test for interactions assessed at both follow-up assessments between the treatment arm and the following variables: sex; age; educational level; comorbid physical illness (diabetes, hypertension, or both); and baseline PHQ-9 levels.

#### Complier Average Causal Effect analyses

Complier Average Causal Effect (CACE) analysis^28^ using an instrumental variable estimator and imputed data was applied to estimate the effect of the number of messages electronically recorded as ‘opened’ on depression outcomes. The protocol^21^ and the Statistical Analysis Plan pre-specified the ‘minimum therapeutic dose’ as listening to “most of the messages”, as it was initially based on self-reported categories (“none”, “a few”, “at least half”, “most of the messages”, and “all of the messages”) of a question from the first follow-up assessment. In the event, we were able to access the electronic data, and we therefore operationalised the pre-specified minimum therapeutic dose as ‘opened’ 36 or more messages (75% of the total of 48 messages) versus ‘opened’ 35 or fewer messages. The CACE analyses were conducted using PHQ-9 score at three and five months, adjusting for stratification. We also conducted sensitivity analyses using thresholds of having ‘opened’ at least half of the total messages (24+) versus 23 messages or fewer and having ‘opened’ all 48 messages versus not doing so.

#### Sensitivity analysis

Any baseline variables imbalanced substantially between treatment arms (assessed using descriptive statistics) were adjusted in a secondary analysis for the primary outcome. We also performed a sensitivity analysis for the primary outcome adjusting for the time between randomisation and the primary follow-up assessment at three months. For both these sensitivity analyses, if there was a noticeable impact we planned to conduct similar analyses for the secondary outcomes.

Regression diagnostics were run for both the linear and Poisson regression models. Normality assumptions for all linear regression models were evaluated by examining the residual plots. The Stata post estimation command *gof*, was used to assess the Poisson model.

Statistical tests were two-sided and all analyses were conducted using Stata 17 (StataCorp). The trial was registered with the ReBEC, RBR-6c7ghfd.

### Role of the funding source

The study funders had no role in study design, data collection, data analysis, data interpretation, or writing of the report.

## Results

**Fig. 1:**
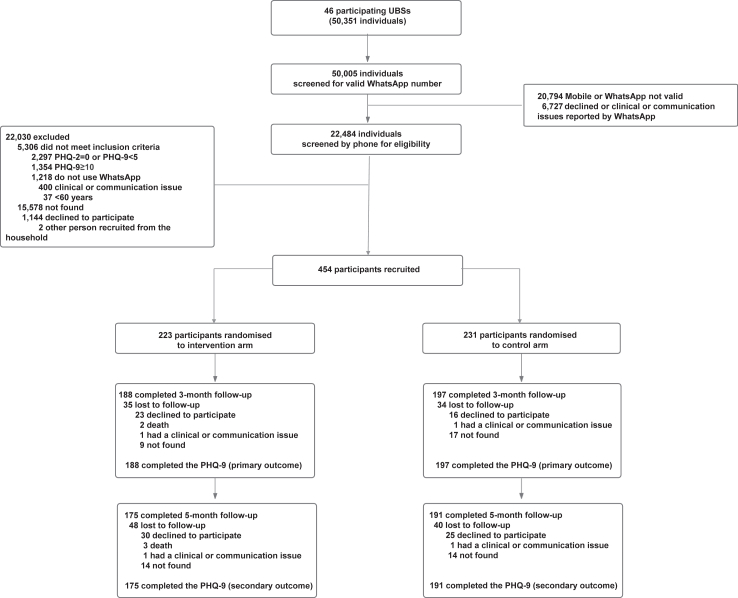
**Trial profile**.

Recruitment started on 8 September 2021 and ended on 9 August 2022, with follow-up assessments completed on 30 January 2023. A total of 50,351 individuals registered with 46 UBSs were entered in the REDCap system. Among the 22,484 (44.7%) who had a valid phone and WhatsApp access and were randomly screened for eligibility by a phone call, 5,306 (23.6%) individuals did not meet the inclusion criteria (of which 3,651 did not present subthreshold depressive symptomatology), 1,144 (5.1%) declined to participate, two (0.01%) lived in the same household as another participant of the randomised controlled trial, and 15,578 (69.3%) were not found after at least two contact attempts. The remaining 454 participants were recruited with 223 (49.1%) randomised to the intervention arm and 231 (50.9%) to the control arm (Fig. 1). A total of 385 (84.8%) of the recruited participants were followed up at three months. Similar proportions of participants were lost to follow-up in the intervention (n = 35, 15.7%) and control arms (n = 34, 14.7%). At five months, 366 (80.6%) participants were followed up, with a greater proportion lost to follow-up in the intervention arm (n = 48, 21.5%) compared with the control (n = 40, 17.3%) arm.

Descriptive statistics of baseline characteristics indicate that most of the participants were 60–69-year-old women, of whom the majority had less than eight years of education and received the minimum wage (Table 1). Findings reported in this table indicate some potential imbalances between arms with, for instance, slightly more participants in the control arm reporting hypertension and diabetes, and slightly fewer reporting taking antidepressants.

**Table 1:** Baseline characteristics in the intervention and control arms.

	Intervention group (n = 223)	Control group (n = 231)
**Sex**		
Male	80 (35.9%)	82 (35.5%)
Female	143 (64.1%)	149 (64.5%)
**Age group, years**		
60–69	181 (81.2%)	183 (79.2%)
70+^a^	42 (18.8%)	48 (20.8%)
**Type of UBS**		
Family Health Strategy	191 (85.7%)	195 (84.4%)
Mixed models	32 (14.4%)	36 (15.6%)
**Education, years**		
None	26 (11.7%)	35 (15.2%)
1–4 years	48 (21.5%)	64 (27.8%)
5–8 years	53 (23.8%)	47 (20.4%)
>8 years	96 (43.1%)	84 (36.5%)
**Personal income, minimum age**		
Up to 1 MW	123 (56.2%)	118 (51.3%)
>1–2 MW	50 (22.8%)	56 (24.4%)
>2 MW	46 (21.0%)	56 (24.4%)
**Race**		
White	95 (41.3%)	94 (42.3%)
Black	20 (8.7%)	26 (11.7%)
Asian	4 (1.7%)	3 (1.4%)
Mixed	111 (48.3%)	96 (43.2%)
Indigenous	–	3 (1.4%)
**Smoker**	46 (20.6%)	27 (11.7%)
**Hypertension (self-reported)**	139 (62.3%)	156 (67.5%)
**Diabetes (self-reported)**	68 (30.5%)	83 (35.9%)
**Receiving pharmacological treatment for depression (self-reported)**	14 (6.3%)	11 (4.8%)
**PHQ-9 scores (SD)**	7.23 (1.36)	6.85 (1.43)
**GAD-7 scores (SD)**	9.33 (4.03)	8.98 (4.17)
**EQ-5D-5L scores (SD)**	0.874 (0.109)	0.901 (0.087)
**ICECAP-O scores (SD)**	0.717 (0.147)	0.729 (0.142)
**3-item UCLA scores (SD)**	4.97 (1.73)	4.94 (1.60)

Abbreviations: 3-item UCLA scores: 3-item University of California, Los Angele loneliness scale; EQ-5D-5L: 5-level EuroQol health-related quality of life questionnaire; GAD-7: 7-item General Anxiety Disorder questionnaire; ICECAP-O: ICEpop CAPability measure for older people; MW: minimum wage (in 2021, the minimum wage in Brazil was BRL1110 (approximately US$213); PHQ-9: 9-item Patient Health Questionnaire; SD: standard deviation; UBS: Unidade Básica de Saúde (primary care clinic).

PHQ-9 scores range from 0 to 27, with higher scores representing more severe depressive symptomatology.

GAD-7 scores range from 0 to 21 with higher scores representing more severe anxiety symptomatology.

EQ-5D-5L scores range from −0.264 to 1, with higher scores representing higher quality of life.

ICECAP-O scores range from 0 to 1, with higher scores representing greater levels of capability wellbeing.

3-item UCLA scores range from 3 to 9 with higher scores representing greater levels of loneliness.

a Participants aged 80+ in the intervention (n = 2) and control arms (n = 5).

Sixteen participants (7.2%) in the intervention arm and ten (4.3%) in the control arm reported hospitalisation not related to mental health during the trial. Three (1.3%) deaths were reported, all in the intervention arm. None of the events were considered related to trial participation.

### Primary and secondary outcomes

The results from the analysis of the primary outcome demonstrated no substantial difference in mean PHQ-9 scores at three months in the intervention arm (6.81 [SD: 5.48]) compared with the control arm (7.10 [SD: 5.62]) (Table 2). The adjusted difference in mean PHQ-9 scores after imputing missing values was −0.61 (95% confidence interval (CI): −1.75, 0.53; *p* = 0.29), and in terms of units of standard deviation all values in the CI are below our target threshold of 0.33. At five months and after imputing missing data, the adjusted difference in mean PHQ-9 scores in the intervention (5.87 [SD: 5.70]) versus the control arm (6.02 [SD: 5.80]) also demonstrated a lack of effect of the intervention (−0.49 [95% CI: −1.67, 0.69]; *p* = 0.41). These findings were similar to those from the complete case analysis (Supplementary Tables S6 and S7).

**Table 2:** Differences in mean PHQ-9 scores between intervention and control arms at the three and five months (primary and secondary outcome respectively).

Outcome	Three-month follow-up				Five-month follow-up			
	Intervention group	Control group	Coefficient (95% CI)^a^^,^^b^	*p* value	Intervention group	Control group	Coefficient (95% CI)^a^^,^^b^	*p* value
**PHQ-9, Number**	188	197			175	191		
Mean (SD)	6.81 (5.48)	7.10 (5.62)	−0.61 (−1.75, 0.53)	0.29	5.87 (5.70)	6.02 (5.80)	−0.49 (−1.67, 0.69)	0.41

*Abbreviations:* CI: confidence interval; PHQ-9: 9-item Patient Health Questionnaire; SD: standard deviation.

PHQ-9 scores range from 0 to 27, with higher scores representing more severe depressive symptomatology.

a Difference in means between treatment arms were estimated using linear regression models, adjusted for stratified variables (sex, age-group, type of UBS), and baseline PHQ-9 score.

b All estimates had missing data imputed separately, by trial arm, using MICE models that included predictors of missingness (Supplementary material), stratification (sex, age-group, type of UBS), baseline PHQ-9 scores, and any imbalances in missingness between treatment arms.

There was no evidence of effects of the intervention on any of the secondary outcomes including proportion of participants with depressive symptomatology (PHQ-9 scores≥10), anxiety symptomatology (GAD-7), health-related quality of life (EQ-5D-5L), capability wellbeing (ICECAP-O), and loneliness (3-item UCLA) in both the complete case analyses (Supplementary Tables S6–S8) and using imputed data (Table 3, Table 4).

**Table 3:** Relative risk of depressive symptomatology (PHQ-9 scores≥10) in the intervention arm, compared to the control arm at the three- and five-month follow-up visits.

	Intervention group^a^	Control group^a^	Relative risk (95% CI)^b^^,^^c^	*p* value
Depressive symptomatology (PHQ-9≥10) at three months	60/188 (31.9%)	61/197 (31.0%)	0.94 (0.71, 1.26)	0.69
Depressive symptomatology (PHQ-9≥10) at five months	34/175 (19.4%)	45/191 (23.6%)	0.73 (0.49, 1.10)	0.14

*Abbreviations:* CI: confidence interval; PHQ-9: 9-item Patient Health Questionnaire.

a The secondary outcomes of depressive symptomatology was defined as PHQ-9 scores greater than or equal to 10 (yes/no).

b Relative risks and 95% CIs were calculated using fixed effects Poisson regression models with log link function and robust error variances, adjusted for the stratified variable (sex, age-group, type of UBS), and baseline PHQ-9 score.

c All estimates had missing data imputed by trial arm using MICE models that included stratification (sex, age-group, and type of UBS), baseline PHQ-9 scores, predictors of missingness, and any imbalances in missingness between treatment arms (Supplementary material).

**Table 4:** Secondary outcomes including anxiety symptomatology (GAD-7), health-related quality of life (EQ-5D-5L), capability wellbeing in older adults (ICECAP-O), and perceived loneliness (3-item UCLA), at the three and five months.

Outcome	Three-month follow-up				Five-month follow-up			
	Intervention group	Control group	Coefficient (95% CI)^a^^,^^b^	*p* value	Intervention group	Control group	Coefficient (95% CI)^a^^,^^b^	*p* value
**GAD-7, number**	188	197			175	191		
Mean (SD)	6.29 (5.60)	6.58 (5.46)	−0.59 (−1.70, 0.51)	0.29	6.15 (5.52)	5.89 (5.57)	−0.02 (−1.09, 1.05)	0.97
Median (IQR)	5 (1–11)	6 (2–10)			5 (2–10)	4 (1–9)		
**EQ-5D-5L, number**	186	197			175	191		
Mean (SD)	0.882 (0.116)	0.900 (0.107)	−0.003 (−0.022, 0.016)	0.78	0.904 (0.102)	0.911 (0.099)	0.007 (−0.010, 0.025)	0.42
Median (IQR)	0.916 (0.849–0.970)	0.927 (0.869–0.970)			0.927 (0.873–0.970)	0.944 (0.883–0.970)		
**ICECAP-O, number**	185	196			175	191		
Mean (SD)	0.726 (0.153)	0.723 (0.148)	0.010 (−0.019, 0.036)	0.53	0.736 (0.149)	0.735 (0.154)	0.012 (−0.016, 0.040)	0.40
Median (IQR)	0.724 (0.620–0.838)	0.724 (0.631–0.828)			0.734 (0.633–0.847)	0.737 (0.606–0.856)		
**3-item UCLA, number**	187	197			175	191		
Mean (SD)	4.75 (1.79)	4.91 (1.87)	−0.28 (−0.58 0.03)	0.072	4.64 (1.75)	4.58 (1.72)	0.03 (−0.30, 0.35)	0.86
Median (IQR)	5 (3–6)	5 (3–6)			4 (3–6)	4 (3–6)		

*Abbreviations:* 3-item UCLA scores: 3-item University of California, Los Angele loneliness scale; EQ-5D-5L: 5-level EuroQol health-related quality of life questionnaire; GAD-7: 7-item General Anxiety Disorder questionnaire; ICECAP-O: ICEpop CAPability measure for older people; IQR: interquartile range; SD: standard deviation.

GAD-7 scores range from 0 to 21 with higher scores representing more severe anxiety symptomatology.

EQ-5D-5L scores range from −0.264 to 1, with higher scores representing higher quality of life.

ICECAP-O scores range from 0 to 1, with higher scores representing greater levels of capability wellbeing.

3-item UCLA scores range from 3 to 9 with higher scores representing greater levels of loneliness.

a Difference in means were estimated using linear regression models, adjusted for the baseline assessment of the corresponding outcome and the stratified variable (sex, age-group, and type of UBS).

b All estimates had missing data imputed separately, by trial arm, using MICE models that included predictors of missingness (Supplementary material), stratification (sex, age-group, type of UBS), corresponding baseline value of outcome in question, and any imbalances in missingness between treatment arms.

### Subgroup analyses

Findings from our subgroup analyses (Supplementary Table S9) revealed that there was no evidence of interactions for any of our pre-specified variables.

### CACE analyses

The electronic system indicated that 177 (79.4%) participants in the intervention arm ‘opened’ at least 36 of the 48 messages (75% of the total messages; our pre-specified minimum therapeutic dose). Estimates from the upper and lower thresholds of our sensitivity analyses indicate that 186 (83.4%) participants ‘opened’ at least 24 messages (50% of the total messages), and 132 (59.2%) participants ‘opened’ all 48 messages. At baseline, participants were asked if they needed help to use WhatsApp, and 312 (68.7%) answered ‘No’, 119 (26.2%) answered ‘Sometimes’, and only 23 (5.1%) answered ‘Always’.

Estimates from the CACE analysis (Table 5) evaluating the effect of opening at least 36 messages (the pre-specified ‘minimum therapeutic dose’) compared with participants opening less than this amount did not show an improvement in mean PHQ-9 scores, at both three-month (adjusted difference between means: −0.93 [95% CI: −2.32, 0.46]; *p* = 0.19) and five-month assessments (−0.74 [95% CI: −2.22, 0.74]; *p* = 0.33). The sensitivity analyses testing upper and lower limits of opening messages resulted in the same conclusions (Table 5).

**Table 5:** Complier Average Causal Effects (CACE) analyses on the mean PHQ-9 scores at three- and five-month follow-ups using electronic recordings.

Threshold	Three-month follow-up		Five-month follow-up	
	Adjusted difference in mean PHQ-9 (95% CI)^a^^,^^b^	*p* value	Adjusted difference in mean PHQ-9 (95% CI)^a^^,^^b^	*p* value
Opened at least 24 messages (n = 186, 83.4%)	−0.89 (−2.21, 0.44)	0.19	−0.61 (−2.02, 0.79)	0.39
Opened at least 36 messages—the minimum therapeutic dose (n = 177, 79.4%)	−0.93 (−2.32, 0.46)	0.19	−0.74 (−2.22, 0.74)	0.33
Opened all 48 messages (n = 132, 59.2%)	−1.24 (−3.10, 0.62)	0.19	−0.98 (−2.96, 0.99)	0.33

*Abbreviations:* CI: confidence interval; PHQ-9: 9-item Patient Health Questionnaire.

PHQ-9 scores range from 0 to 27, with higher scores representing more severe depressive symptomatology.

a All models are adjusted for stratification (sex, age-group, type of UBS), and baseline PHQ-9 scores.

b All estimates had missing data imputed separately, by trial arm, using MICE models that included predictors of missingness (Supplementary material), stratification (sex, age-group, type of UBS), and baseline PHQ-9 scores. Any imbalances in missingness were also included in MICE models.

### Sensitivity analyses

Given the imbalances between the treatment arms at baseline (Table 1), we ran a sensitivity analysis adjusting liberally for them as well as stratification factors that were accounted for in the primary analyses. This sensitivity analysis for our primary outcome suggests that estimates shifted towards the null, but the 95% CIs still overlapped substantially with those from the primary analysis (Supplementary Table S10). Adjusting for the number of days between randomisation and the first follow-up assessment led to no appreciable impact on the results for the primary outcome (Supplementary Material, page 13), and hence neither of these sensitivity analyses were extended to the secondary outcomes. The sensitivity analyses checking for maintenance of randomisation at three and five months can be found in the Supplementary Tables S11 and S12.

## Discussion

To the best of our knowledge Viva Vida is the first digital self-help psychosocial intervention aiming to manage subthreshold depressive symptoms among older adults in Low- and Middle-Income Countries. Viva Vida delivered automated messages without any support from healthcare workers. The present study found no evidence of any improvement in subthreshold depressive symptoms post intervention among older adults at three and five months compared with controls. Nor did Viva Vida prevent participants from presenting depressive symptomatology at either follow-up assessments. In addition, this study found no evidence of improvements in any of our other secondary outcomes (anxiety symptomatology, health-related quality of life, capability wellbeing, and perceived loneliness).

Previous studies had shown evidence of the effectiveness of other digital interventions for subthreshold depression among adults and older adults.^29^ However, most of these studies offered some kind of support or contact with a health professional and the majority were conducted in high-income countries. We identified only two digital interventions without professional support for individuals with subthreshold depression, but both included younger people, and the content and techniques differed from PRODIGITAL. One study assessing an eight-week intervention based on cognitive behavioural therapy found a moderate effect size in participants 50+ years (mean age of 55 years) in the Netherlands.^30^ Another study delivered automated emails with self-help behaviour strategies (Mood Memos) over six weeks to adults (mean age 36 years) in Australia.^31^ This intervention showed a small effect size in reducing PHQ-9 scores but no differences in the risk of major depression. Both studies showed a higher loss to follow-up rate (34–59%) for the primary outcome in the digital intervention arm compared with the 16% rate observed in our study.

One potential explanation for our null findings is the absence of contact with professionals or lay groups, which has been reported as an important feature in a systematic review of digital interventions for older adults.^8^ Our messages were automatically delivered, whereby participants only interacted with a ‘quick reply’ tool. However, lack of human interaction may not be the only explanation for our null findings. According to an individual participant data meta-analysis of randomised controlled trials, guided digital interventions showed higher, though not statistically significant, effects compared with unguided interventions for subthreshold depression in adults.^29^ Another network meta-analysis with individual patient data also found small difference in outcomes between guided and unguided interventions.^9^ Another possible explanation for our findings is related to the proportion of audio messages sent to participants. Specifically, participants in the present study received a version of the Viva Vida intervention with fewer audio messages (50% of all messages) and more visual messages than the version of the Viva Vida used for treating depression^18^ in older adults (83% of all messages). Given the latter intervention demonstrated an effective reduction in depressive symptoms at three months,^18^ it is possible that a slightly more intense programme may be better suited for reducing subthreshold depressive symptoms.

This Viva Vida digital programme was especially tailored to the target population, considering low levels of education and socioeconomic conditions, to ensure the acceptability, appropriateness, and feasibility of the programme that is delivered and received with high fidelity. To accommodate those with lower literacy levels, we incorporated audio and visual messages with minimal written content. Moreover, the programme's characters used an appropriate and simple language to share stories that older adults can identify with, encouraging reflection on similar life situations. Simpler and user-friendly technologies were identified by the previously described systematic review as another important feature of successful digital intervention for older adults.^8^ WhatsApp is the most used messaging application in Brazil, and it is popular among this age group.^16^ This design choice eliminated the need for participants to download new applications or acquire additional digital skills. We believe these elements contributed to the lower than anticipated loss to follow-up rate and the high proportion of ‘opened’ messages. Future studies should explore the potential benefits of personalised approaches in such interventions, such as sex/gender, relevant socioeconomic or clinical characteristics (for example, education level or comorbidities), and individual preferences.

The lack of a well-established standardised criterion to capture subthreshold depression is an important consideration for future research. While this study assessed subthreshold depressive symptoms using a validated screening scale that could be easily used in primary care, other studies assessing psychosocial interventions for older adults with subthreshold depression used standardised clinical assessments.^27^^,^^32^ Additionally, unlike those studies that utilised the PHQ-9 score or other validated screening scales solely to assess the outcome measure, we employed the same instrument for both eligibility criteria and the primary outcome assessment. Given that initial symptom severity is known to influence reduction in symptoms of depression,^33^ it is difficult to compare findings with other studies. Consideration is needed to standardise definitions of subthreshold depression, so that results from different trials are comparable.

This study has limitations. An important consideration is our system's ability to accurately monitor adherence to the intervention. The WhatsApp Business Application Processing Interface provided information indicating if/when the chat window was opened, but this does not provide a measure of engagement with the messages. Moreover, this timestamp was only recorded if the feature was not turned off by the participant. Therefore, it was not possible to evaluate the actual adherence (listening to the messages) to the intervention. Importantly, recruitment was conducted over the phone following a list provided by the Guarulhos Health Secretary. Strategies were implemented to ensure credibility and reassure individuals that the study was authentic, such as placing posters at the UBSs, and instructing UBS staff to confirm the study details. Individuals were also notified about the upcoming call via text message. Despite these efforts, most of the individuals were excluded due to non-response. In Brazil, changes in phone numbers or temporary periods of inactivity are common, which may also have contributed to the low recruitment yield rate. Lastly, as with other digital interventions, it is possible that the trial may have unintentionally created a digital divide whereby the most socioeconomically vulnerable individuals were excluded since access to WhatsApp was one inclusion criterion. Future trials could also consider providing a pool of smartphones to ensure vulnerable participants are not excluded but penetration of these phones is high in Brazil.

Another limitation of Viva Vida is our inability to monitor behaviour changes. Although we were able to monitor whether messages were opened, we do not know if they were read or if it translated to behaviour change. This study was conducted during the COVID-19 pandemic and we were unable to conduct face-to-face interviews but rather the interviews were made by phone. This limited the number of questions we could ask and observations made. In a previous study,^15^ research assistants found that they were unable to administer the Behavioural Activation for Depression Scale^34^ over the telephone without the use of visual aids, a challenge they did not encounter during face-to-face interviews.

Given the importance of understanding whether the Viva Vida self-help intervention effectively changed behaviour, future research should explore how to best capture behavioural changes in this population via telephone interviews. Another possibility to approach this is to use Viva Vida's ‘quick reply’ tool during the intervention to assess whether the audio messages have motivated participants and led to behaviour changes, although this remains a self-reported measure. Additionally, since previous research suggests that human support is an important feature of digital interventions in older adults,^8^ future studies should consider incorporating low-cost support mechanisms. These could include community support groups via social media or in-person led by peers or individuals with lived experience. Such groups could be part of a stepped-care strategy to improve subthreshold depressive symptoms and prevent the development of depression in older adults.

## Contributors

CAN, TJP, WH, RA and MS conceptualised the study. TJP, WH, RA and MS acquired funding. CAN, TVDN, RA and MS contributed to project administration. CAN, TVDN, FAM, TJP, RA and MS supervised the trial. All authors contributed to the methodology. CAN, TVND, FAM, MOC, CHQS, GMO, MSS, LAP, MMSM and MS contributed to the investigation. CAN, NS, FAM, MOC, CHQS, and GMO curated the data. NS and TJP contributed to formal data analysis. FAM, MOC, CHQS, GMO and PVV developed and maintained the web application. CAN, NS, TJP, RA and MS wrote the original draft. All authors reviewed and edited the manuscript. CAN and NS have accessed and verified the data. CAN, NS, TJP, RA and MS had full access to all the data in the study and had final responsibility for the decision to submit for publication.

## Data sharing statement

De-identified individual participant data and a data dictionary will be made available 24 months after publication. Proposals with specific aims and an analysis plan should be directed to the corresponding author.

## Editorial disclaimer

The translation of the Summary was submitted by the authors, and we reproduce it as supplied. It has not been peer reviewed. Our editorial processes have only been applied to the original version in English, which should serve as a reference for this manuscript.

## Declaration of interests

We declare no competing interests.

## Appendix A Supplementary data

Supplementary Tables

Protocol

Summary Portuguese
