# Supplementary material for: A self-help mobile messaging intervention to improve subthreshold depressive symptoms among older adults in a socioeconomically deprived region of Brazil (PRODIGITAL): a pragmatic, two-arm randomised controlled trial
Source: Lancet Reg Health Am. 2024 Oct 7;39:100897. doi: 10.1016/j.lana.2024.100897 (PMC11491721; doi:10.1016/j.lana.2024.100897)
Supplement: Supplementary Tables [file mmc1.docx]

A self-help mobile messaging intervention to improve subthreshold depressive symptoms among older adults in a socioeconomically deprived region of Brazil (PRODIGITAL): a pragmatic, two-arm randomised controlled trial

[Severe adverse events 1](#_Toc176745377)

[Supplementary Table 1: Cause of hospitalisation collected at three and five months 1](#_Toc176745378)

[Sensitivity analyses - missing data analyses 2](#_Toc176745379)

[Supplementary Table 2: Comparison between participants with complete data and participants with missing data at three months 3](#_Toc176745380)

[Supplementary Table 3: Comparison between participants with complete data and participants with missing data at five months 4](#_Toc176745381)

[Supplementary Table 4: Model 1 - Adjusted risk ratio for presenting depressive symptomatology (PHQ-9 scores≥10) at three months for different departures from the missing at random assumption, assuming greater probability of the outcome being missing when depressive symptomatology was present 5](#_Toc176745382)

[Supplementary Table 5: Model 2 - Adjusted risk ratio for presenting depressive symptomatology (PHQ-9 scores≥10) at five months for different departures from the missing at random assumption, assuming greater probability of the outcome being missing when depressive symptomatology was present 6](#_Toc176745383)

[Results comparing estimates from MICE models with complete case analyses for both primary and secondary outcomes 7](#_Toc176745384)

[Supplementary Table 6: Comparison of estimates from multiple imputed data using MICE models, with estimates from complete case analysis for the primary outcome of difference in mean PHQ-9 scores between treatment arms and difference in mean of other continuous secondary outcomes at three months 7](#_Toc176745385)

[Supplementary Table 7: Comparison of estimates from multiple imputed data using MICE models, with estimates from complete case analysis for secondary outcomes at five months 8](#_Toc176745386)

[Supplementary Table 8: Comparison of adjusted relative risks from multiple imputed data using MICE models, with estimates from complete case analysis for the secondary outcomes of depressive symptomatology at three and five months 9](#_Toc176745387)

[Subgroup analyses – moderation by pre-specified variables for both the primary or secondary outcomes 10](#_Toc176745388)

[Supplementary Table 9: Results of Wald test for models testing for interaction terms with treatment allocations at three and five months with the outcome of difference in mean PHQ-9 scores 10](#_Toc176745389)

[Sensitivity analyses testing for influence of baseline imbalances on the primary outcome of difference in mean PHQ-9 scores 11](#_Toc176745390)

[Supplementary Table 10: Sensitivity analyses estimating the effect of adjusting for imbalances in randomisation, on the estimates for the primary outcome (difference in mean PHQ-9 scores at three months) using complete data only 11](#_Toc176745391)

[Sensitivity analyses checking for maintenance of randomisation (complete cases only) 12](#_Toc176745392)

[Supplementary Table 11: Comparison of baseline demographics and baseline measures for secondary outcomes, between trial arms, for participants followed-up at three months 12](#_Toc176745393)

[Supplementary Table 12: Comparison of baseline demographics and baseline measures for secondary outcomes, between trial arms, for participants followed-up at five months 13](#_Toc176745394)

Severe adverse events

Supplementary Table 1: Cause of hospitalisation collected at three and five months

|  | **Intervention group (n=16)** | **Control group (n=10)** |
| --- | --- | --- |
| Cardiovascular and metabolic conditions | 1 | 3 |
| Respiratory conditions | 2 | 0 |
| Neurological conditions | 0 | 1 |
| Blood disorders | 1 | 2 |
| Urological conditions | 1 | 0 |
| Surgical procedures | 11 | 3 |
| Trauma | 0 | 1 |

Sensitivity analyses - missing data analyses

Supplementary Methods

Initially, patterns of missingness were investigated by comparing missing with complete data for the baseline demographic characteristics and morbidity questionnaires with mean PHQ-9 scores, by trial arm, at both follow-up assessments. In order to reduce bias and loss of information, we used multiple imputation by chained equations (MICE) with 50 imputations, as implemented in the MI command in Stata under the assumption that data were missing at random (MAR).^1^ Variables used in the MICE models consisted of the outcome of PHQ-9 scores, covariates described in the Statistical Analysis Plan (age group, sex, type of Unidade Básica de Saúde (UBS, primary care clinic), baseline PHQ-9 score, and treatment arm) and variables found to be predictors of missingness.^2,3^ Predictors of missingness include the following: education, employment, health-related quality of life (EQ-5D-5L) at baseline, and tobacco use at baseline. Potential predictors that theoretically could have influenced levels of missingness include presence of hypertension, tobacco use, and levels of enjoyable activities participants engaged in. Baseline scores for corresponding outcome models including depressive symptomatology (PHQ-9), anxiety symptomatology (GAD-7), capability wellbeing (ICECAP-O), and levels of loneliness (3-item UCLA) were also included.

To assess the sensitivity of our findings against modest departures from the MAR assumption, a weighted sensitivity analysis using the Selection Model Approach was applied.^4-6^ Briefly, once data had been imputed under MAR, parameter estimates from each imputed dataset were reweighted to allow for the data to be missing not at random (MNAR). To test the stability of our model, we considered different degrees of departure from the MAR assumption by considering plausible values of δ ranging from 0·10 to 0·40. This range corresponds to relative risk for the data being observed when a participant presented depressive symptomatology (PHQ-9 scores≥10) compared to when it did not, ranging from 1·11 to 1·50 (that is, the exponential of 0·10 and 0·40 respectively).

Results

General

Supplementary Tables 2 and 3 demonstrate findings from the analysis comparing baseline demographics and baseline measures for the secondary outcomes between participants with complete data, and those with missing data, at three- and five-months follow-up assessments respectively.

Supplementary Table 2: Comparison between participants with complete data and participants with missing data at three months

|  | **Intervention group** | | **Control group** | |
| --- | --- | --- | --- | --- |
|  | **Total (n=188)** | **Missing PHQ-9 (n=35)** | **Total (n=197)** | **Missing PHQ-9 (n=34)** |
| **Sex, No. (%)** |  |  |  |  |
| Male | 66 (35·1%) | 14 (40·0%) | 72 (36·6%) | 10 (29·4%) |
| Female | 122 (64·9%) | 21 (60·0%) | 125 (63·5%) | 24 (70·6%) |
| **Age group, No. (%)** |  |  |  |  |
| 60-69 years | 148 (78·7%) | 33 (94·3%) | 158 (80·2%) | 25 (73·5%) |
| 70+ years | 40 (21·3%) | 2 (5·7%) | 39 (19·8%) | 9 (26·5%) |
| **Education, No. (%)** | 172 (91·5%) | 31 (88·6%) | 170 (86·3%) | 32 (94·1%) |
| **Education (level), No. (%)** |  |  |  |  |
| None | 21 (11·2%) | 5 (14·3%) | 32 (16·3%) | 3 (8·8%) |
| 1-4 years | 38 (20·2%) | 10 (28·6%) | 54 (27·6%) | 10 (29·4%) |
| 5-8 years | 46 (24·5%) | 7 (20·0%) | 36 (18·4%) | 11 (32·4%) |
| >8 years | 83 (44·2%) | 13 (37·1%) | 74 (37·8%) | 10 (29·4%) |
| **Personal income, No. (%)** |  |  |  |  |
| Up to 1 MW | 107 (57·5%) | 16 (48·5%) | 100 (51·0%) | 18 (52·9%) |
| >1-2 MW | 40 (21·5%) | 10 (30·3%) | 48 (24·5%) | 8 (23·5%) |
| >2 MW | 39 (21·0%) | 7 (21·2%) | 48 (24·5%) | 8 (23·5%) |
| **Household income, No. (%)** |  |  |  |  |
| Up to 1 MW | 56 (31·1%) | 8 (25·0%) | 46 (23·8%) | 6 (18·8%) |
| >1-2 MW | 44 (24·4%) | 10 (31·3%) | 60 (31·1%) | 13 (40·6%) |
| >2 MW | 80 (44·4%) | 14 (43·8%) | 87 (45·1%) | 13 (40·6%) |
| **Job, No. (%)** | 69 (36·7%) | 14 (40·0%) | 68 (34·5%) | 14 (41·2%) |
| **Smoker, No. (%)** | 32 (17·0%) | 14 (40·0%) | 24 (12·2%) | 3 (8·8%) |
| **Hypertension (self-reported), No. (%)** | 114 (60·6%) | 25 (71·4%) | 134 (68·0%) | 22 (64·7%) |
| **Diabetes (self-reported), No. (%)** | 58 (30·9%) | 10 (28·6%) | 72 (36·6%) | 11 (32·4%) |
| **Mean PHQ-9 scores (SD)** | 7·29 (1·35) | 6·94 (1·41) | 6·87 (1·43) | 6·76 (1·46) |
| **Mean GAD-7 scores (SD)** | 9·43 (4·12) | 8.73 (3.48) | 8·89 (4·16) | 9·54 (4·28) |
| **Mean EQ-5D-5L scores (SD)** | 0·880 (0·098) | 0.842 (0.154) | 0·901 (0·086) | 0·896 (0·095) |
| **Mean ICECAP-O scores (SD)** | 0·716 (0·146) | 0.718 (0.159) | 0·729 (0·145) | 0·730 (0·121) |
| **Mean 3-item UCLA scores (SD)** | 5·02 (1·74) | 4.74 (1.72) | 4·89 (1·65) | 5·24 (1·33) |
| **Engagement in enjoyable, meaningful activities over the past two weeks, No. (%)** |  |  |  |  |
| Not at all | 34 (18·1%) | 3 (8·6%) | 29 (14·8%) | 4 (11·8%) |
| Several days | 32 (17·0%) | 8 (22·9%) | 33 (16·8%) | 5 (14·7%) |
| Over half the days | 31 (16·5%) | 6 (17·1%) | 38 (19·4%) | 5 (14·7%) |
| Nearly every day | 91 (48·4%) | 18 (51·4%) | 96 (49·0%) | 20 (58·8%) |

*Abbreviations:* 3-item UCLA scores: 3-item University of California, Los Angele loneliness scale; EQ-5D-5L: 5-level EuroQol health-related quality of life questionnaire; GAD-7: 7-item General Anxiety Disorder questionnaire; ICECAP-O: ICEpop CAPability measure for older people; MW: minimum wage (in 2021, the minimum wage in Brazil was BRL1110 (approximately US$213); PHQ-9: 9-item Patient Health Questionnaire; SD: standard deviation

Supplementary Table 3: Comparison between participants with complete data and participants with missing data at five months

|  | **Intervention group** | | **Control group** | |
| --- | --- | --- | --- | --- |
|  | **Total (n=175)** | **Missing PHQ-9 (n=48)** | **Total (n=191)** | **Missing PHQ-9 (n=40)** |
| **Sex, No. (%)** |  |  |  |  |
| Male | 63 (36·0%) | 17 (35·4%) | 68 (35·6%) | 14 (35·0%) |
| Female | 112 (64·0%) | 31 (64·6%) | 123 (64·4%) | 26 (65·0%) |
| **Age group, No. (%)** |  |  |  |  |
| 60-69 years | 140 (80·0%) | 41 (85·4%) | 155 (81·2%) | 28 (70·0%) |
| 70+ years | 35 (20·0%) | 7 (14·6%) | 36 (18·8%) | 12 (30·0%) |
| **Education, No. (%)** | 161 (92·0%) | 42 (87·5%) | 164 (85·9%) | 38 (95·0%) |
| **Education (level), No. (%)** |  |  |  |  |
| None | 19 (10·9%) | 7 (14·6%) | 32 (16·8%) | 3 (7·5%) |
| 1-4 years | 35 (20·0%) | 13 (27·1%) | 50 (26·3%) | 14 (35·0%) |
| 5-8 years | 42 (24·0%) | 11 (22·9%) | 36 (19·0%) | 11 (27·5%) |
| >8 years | 79 (45·1%) | 17 (35·4%) | 72 (37·9%) | 12 (30·0%) |
| **Personal income, No. (%)** |  |  |  |  |
| Up to 1 MW | 96 (55·5%) | 27 (58·7%) | 103 (54·2%) | 15 (37·5%) |
| >1-2 MW | 37 (21·4%) | 13 (28·3%) | 44 (23·2%) | 12 (30·0%) |
| >2 MW | 40 (23·1%) | 6 (13·0%) | 43 (22·6%) | 13 (32·5%) |
| **Household income, No. (%)** |  |  |  |  |
| Up to 1 MW | 49 (29·2%) | 15 (34·1%) | 45 (24·1%) | 7 (18·4%) |
| >1-2 MW | 39 (23·2%) | 15 (34·1%) | 59 (31·6%) | 14 (36·8%) |
| >2 MW | 80 (47·6%) | 14 (31·8%) | 83 (44·4%) | 17 (44·7%) |
| **Job, No. (%)** | 66 (37·7%) | 17 (35·4%) | 66 (34·6%) | 16 (40·0%) |
| **Smoker, No. (%)** | 29 (16·6%) | 17 (35·4%) | 23 (12·0%) | 4 (10·0%) |
| **Hypertension (self-reported), No. (%)** | 107 (61·1%) | 32 (66·7%) | 130 (68·1%) | 26 (65·0%) |
| **Diabetes (self-reported), No. (%)** | 53 (30·3%) | 15 (31·3%) | 68 (35·6%) | 15 (37·5%) |
| **Mean PHQ-9 scores (SD)** | 7·26 (1·35) | 7·15 (1·40) | 6·85 (1·44) | 6·85 (1·41) |
| **Mean GAD-7 scores (SD)** | 9·37 (4·07) | 9·17 (3·95) | 8·82 (4·16) | 9·77 (4·20) |
| **Mean EQ-5D-5L scores (SD)** | 0·877 (0·100) | 0·862 (0·139) | 0·902 (0·088) | 0·893 (0·085) |
| **Mean ICECAP-O scores (SD)** | 0·715 (0·146) | 0·722 (0·153) | 0·734 (0·147) | 0·706 (0·112) |
| **Mean 3-item UCLA scores (SD)** | 5·00 (1.75) | 4·88 (1·68) | 4·90 (1·64) | 5·18 (1·43) |
| **Engagement in enjoyable, meaningful activities over the past two weeks, No. (%)** |  |  |  |  |
| Not at all | 34 (19·4%) | 3 (6·3%) | 26 (13·7%) | 7 (17·5%) |
| Several days | 29 (16·6%) | 11 (22·9%) | 33 (17·4%) | 5 (12·5%) |
| Over half the days | 28 (16·0%) | 9 (18·8%) | 37 (19·5%) | 6 (15·0%) |
| Nearly every day | 84 (48·0%) | 25 (52·1%) | 94 (49·5%) | 22 (55·0%) |

*Abbreviations:* 3-item UCLA scores: 3-item University of California, Los Angele loneliness scale; EQ-5D-5L: 5-level EuroQol health-related quality of life questionnaire; GAD-7: 7-item General Anxiety Disorder questionnaire; ICECAP-O: ICEpop CAPability measure for older people; MW: minimum wage (in 2021, the minimum wage in Brazil was BRL1110 (approximately US$213); PHQ-9: 9-item Patient Health Questionnaire; SD: standard deviation

Sensitivity analysis checking the missing at random (MAR) assumption for the secondary outcome

Results from the sensitivity analysis testing the MAR assumption for the secondary outcome of relative risk of depressive symptomatology (PHQ-9 scores≥10) at three and five months suggest that estimates moved away the null.

Supplementary Table 4: Model 1 - Adjusted risk ratio for presenting depressive symptomatology (PHQ-9 scores≥10) at three months for different departures from the missing at random assumption, assuming greater probability of the outcome being missing when depressive symptomatology was present

| **Delta** | **Adjusted relative risk of depressive symptomatology (95% CI)** |
| --- | --- |
| 0·1 | 0·910 (0·586, 1·415) |
| 0·2 | 0·906 (0·585, 1·403) |
| 0·3 | 0·901 (0·584, 1·391) |
| 0·4 | 0·897 (0·583, 1·380) |

*Abbreviations:* CI: confidence interval

Supplementary Table 5: Model 2 - Adjusted risk ratio for presenting depressive symptomatology (PHQ-9 scores≥10) at five months for different departures from the missing at random assumption, assuming greater probability of the outcome being missing when depressive symptomatology was present

| **Delta** | **Adjusted relative risk of depressive symptomatology (95% CI)** |
| --- | --- |
| 0·1 | 0·654 (0·383, 1·119) |
| 0·2 | 0·648 (0·380, 1·107) |
| 0·3 | 0·644 (0·377, 1·101) |
| 0·4 | 0·641 (0·375, 1·100) |

*Abbreviations:* CI: confidence interval

Results comparing estimates from MICE models with complete case analyses for both primary and secondary outcomes

Supplementary Table 6: Comparison of estimates from multiple imputed data using MICE models, with estimates from complete case analysis for the primary outcome of difference in mean PHQ-9 scores between treatment arms and difference in mean of other continuous secondary outcomes at three months

|  | **Complete case analysis^a^** | | **Imputed data from MICE analyses^a,b^** | |
| --- | --- | --- | --- | --- |
| **Model** | **Difference in means (95% CI)** | ***P* Value** | **Difference in means (95% CI)** | ***P* Value** |
| PHQ-9 scores^a^ | -0·64 (-1·75, 0·46) | 0·25 | -0·61 (-1·75, 0·53) | 0·29 |
| GAD-7 scores^a^ | -0·64 (-1·80, 0·52) | 0·28 | -0·59 (-1·70, 0·51) | 0·29 |
| EQ-5D-5L scores^a^ | -0·004 (-0·023, 0·015) | 0·71 | -0·003 (-0·022, 0·016) | 0·78 |
| ICECAP-O scores^a^ | 0·009 (-0·019, 0·036) | 0·55 | 0·010 (-0·019, 0·036) | 0·53 |
| 3-item UCLA scores^a^ | -0·25 (-0·55, 0·05) | 0·11 | -0·28 (-0·58, 0·03) | 0·072 |

*Abbreviations:* 3-item UCLA scores: 3-item University of California, Los Angele loneliness scale; CI: confidence interval; EQ-5D-5L: 5-level EuroQol health-related quality of life questionnaire; GAD-7: 7-item General Anxiety Disorder questionnaire; ICECAP-O: ICEpop CAPability measure for older people; MICE: multiple imputation by chained equations; PHQ-9: 9-item Patient Health Questionnaire

PHQ-9 scores range from 0 to 27, with higher scores representing more severe depressive symptomatology

GAD-7 scores ranges from 0 to 21 with higher scores representing more severe anxiety symptomatology

EQ-5D-5L scores range from -0·264 to 1, with higher scores representing higher quality of life

ICECAP-O scores range from 0 to 1, with higher scores representing greater levels of capability wellbeing

3-item UCLA scores range from 3 to 9, with higher scores representing more severe levels of loneliness

^a^ Differences in means were estimated using linear regression models, adjusted for relevant baseline assessment of corresponding outcome, sex, age-group, type of UBS (full Family Health Strategy or mixed models)

^b^ All estimates had missing data imputed seperately, by trial arm, using MICE models that included predictors of missingness, sex, age-group, type of UBS, and imbalances in missingness between treatment arms, and baseline value of corresponding outcome variable.

Supplementary Table 7: Comparison of estimates from multiple imputed data using MICE models, with estimates from complete case analysis for secondary outcomes at five months

|  | **Complete case analysis^a^** | | **Imputed data from MICE analyses^a,b^** | |
| --- | --- | --- | --- | --- |
| **Model** | **Difference in means (95% CI)** | ***P* Value** | **Difference in means (95% CI)** | ***P* Value** |
| PHQ-9 scores^a^ | -0·52 (-1·70, 0·65) | 0·38 | -0·49 (-1·67, 0·69) | 0·41 |
| GAD-7 scores^a^ | 0·15 (-1·06, 1·35) | 0·81 | -0·02 (-1·09, 1·05) | 0·97 |
| EQ-5D-5L scores^a^ | 0·007 (-0·011, 0·025) | 0·46 | 0·007 (-0·010, 0·025) | 0·42 |
| ICECAP-O scores^a^ | 0·009 (-0·020, 0·038) | 0·54 | 0·012 (-0·016, 0·040) | 0·40 |
| 3-item UCLA scores^a^ | 0·001 (-0·28, 0·28) | 0·99 | 0·03 (-0·30, 0·35) | 0·86 |

*Abbreviations:* 3-item UCLA scores: 3-item University of California, Los Angele loneliness scale; CI: confidence interval; EQ-5D-5L: 5-level EuroQol health-related quality of life questionnaire; GAD-7: 7-item General Anxiety Disorder questionnaire; ICECAP-O: ICEpop CAPability measure for older people; MICE: multiple imputation by chained equations; PHQ-9: 9-item Patient Health Questionnaire

PHQ-9 scores range from 0 to 27, with higher scores representing more severe depressive symptomatology

GAD-7 scores ranges from 0 to 21 with higher scores representing more severe anxiety symptomatology

EQ-5D-5L scores range from -0·264 to 1, with higher scores representing higher quality of life

ICECAP-O scores range from 0 to 1, with higher scores representing greater levels of capability and general wellbeing

3-item UCLA scores range from 3 to 9, with higher scores representing more severe levels of loneliness

^a^ Differences in means were estimated using linear regression models, adjusted for relevant baseline assessment of corresponding outcome, sex, age-group, type of UBS (full Family Health Strategy or mixed models)

^b^ All estimates had missing data imputed seperately, by trial arm, using MICE models that included predictors of missingness, sex, age-group, type of UBS, and baseline value of corresponding outcome measure. Differences in missingness were also included in MICE models.

Supplementary Table 8: Comparison of adjusted relative risks from multiple imputed data using MICE models, with estimates from complete case analysis for the secondary outcomes of depressive symptomatology at three and five months

|  | **Complete case analysis^b,c^** | | **Imputed data from MICE analyses^b,c,d^** | |
| --- | --- | --- | --- | --- |
| **Model** | **Odds ratio (95% CI)** | ***P* Value** | **Odds ratio (95% CI)** | ***P* Value** |
| Depressive symptomatology (PHQ-9≥10) at three months^a,b^ | 0·93 (0·70, 1·24) | 0·63 | 0·94 (0·72, 1·26) | 0·69 |
| Depressive symptomatology (PHQ-9≥10) at five months^a,b^ | 0·74 (0·50, 1·09) | 0·12 | 0·73 (0·49, 1·10) | 0·14 |

*Abbreviations:* CI: confidence interval; MICE: multiple imputation by chained equations; PHQ-9, 9-item Patient Health Questionnaire

^a^ The secondary outcomes of risk of depression was defined as PHQ-9 scores≥10

^b^ Odds ratios and 95% CIs were calculated using Poisson regression models with a loglink function

^c^ Models were adjusted for adjusted for sex, age-group, type of UBS (full Family Health Strategy or mixed models), and baseline PHQ-9 scores.

^d^ All estimates had missing data imputed by trial arm using MICE models that included sex, age-group, type of UBS, baseline PHQ-9 scores and predictors of missingness

Subgroup analyses – moderation by pre-specified variables for both the primary or secondary outcomes

There was evidence the sex moderated the effect of the intervention on the primary and secondary outcomes of difference in mean PHQ-9 scores (Supplementary Table 9). Multiplicative interactions were assessed on the log odds scale.

Supplementary Table 9: Results of Wald test for models testing for interaction terms with treatment allocations at three and five months with the outcome of difference in mean PHQ-9 scores

|  | ***P* Value of Wald test** | |
| --- | --- | --- |
| **Interaction term with treatment allocation** | **Three-month follow-up** | **Five-month follow-up** |
| Sex | 0·055 | 0·096 |
| Age | 0·43 | 0·66 |
| Education | 0·45 | 0·75 |
| Baseline PHQ-9 scores | 0·62 | 0·29 |
| Hypertension | 0·065 | 0·16 |
| Diabetes | 0·21 | 0·92 |
| Comorbid hypertension and diabetes | 0·17 | 0·33 |
| Type of UBS | 0.71 | 0.30 |

*Abbreviations:* PHQ-9: 9-item Patient Health Questionnaire; UBS: Unidade Básica de Saúde (primary care clinic)

Sensitivity analyses testing for influence of baseline imbalances on the primary outcome of difference in mean PHQ-9 scores

Supplementary Table 10: Sensitivity analyses estimating the effect of adjusting for imbalances in randomisation, on the estimates for the primary outcome (difference in mean PHQ-9 scores at three months) using complete data only

| Model adjusted for imbalance | Estimate (95% CI)^a^ | *P* value |
| --- | --- | --- |
| Main model adjusting for sex, age-group, type of UBS, and PHQ-9 scores (baseline) | -0·64 (-1·75, 0·46) | 0·25 |
| 2. Model 1 + education group, hypertension, diabetes, pharmacological treatment for depression (baseline), GAD-7 scores (baseline), EQ-5D-5L scores (baseline) | -0·23 (-1·44, 0·98) | 0·71 |

*Abbreviations:* CI: confidence interval; EQ-5D-5L: 5-level EuroQol health-related quality of life questionnaire; GAD-7: 7-item General Anxiety Disorder questionnaire; PHQ-9: 9-item Patient Health Questionnaire; UBS: Unidade Básica de Saúde (primary care clinic)

PHQ-9 scores range from 0 to 27, with higher scores representing more severe depressive symptomatology

^a^ Difference in means between treatment arms were estimated using linear regression models, adjusted for stratified variables (sex, age-group, type of UBS (full Family Health Strategy or mixed models)), and baseline PHQ-9 scores

Sensitivity analyses checking for maintenance of randomisation (complete cases only)

Sensitivity analysis to determine if randomisation was maintained at three and five months

The comparison at the three-month assessment (Supplementary Table 11) suggests a slight imbalance whereby participants in the intervention arm had higher levels of hypertension and a greater proportion of participants with more severe depressive symptomatology compared with participants in the control arm. Additionally, at the three-month follow-up assessment participants in the control arm received a lower proportion of participants reporting pharmacological treatment for depression and a greater proportion of participants on higher incomes, than participants in the intervention arm. There were also some minor imbalances at the five-month follow-up assessment (Supplementary Table 12). However, none of these observed differences were deemed to be sufficiently large to warrant adjustment in the relevant secondary analyses.

Supplementary Table 11: Comparison of baseline demographics and baseline measures for secondary outcomes, between trial arms, for participants followed-up at three months

|  | **Intervention group (n=188)** | **Control group (n=197)** |
| --- | --- | --- |
| **Sex, No. (%)** |  |  |
| Male | 66 (35·1%) | 72 (36·6%) |
| Female | 122 (64·9%) | 125 (63·5%) |
| **Age group, No. (%)** |  |  |
| 60-69 years | 148 (78·7%) | 158 (80·2%) |
| 70+ years | 40 (21·3%) | 39 (19·8%) |
| **Type of UBS, No. (%)** |  |  |
| Family Health Strategy | 158 (84·0%) | 165 (83·8%) |
| Mixed models | 30 (16·0%) | 32 (16·2%) |
| **Education (level), No. (%)** |  |  |
| None | 21 (11·2%) | 32 (16·3%) |
| 1-4 years | 38 (20·2%) | 54 (27·6%) |
| 5-8 years | 46 (24·5%) | 36 (18·4%) |
| >8 years | 83 (44·2%) | 74 (37·8%) |
| **Personal income, No. (%)** |  |  |
| Up to 1 MW | 107 (57·5%) | 100 (51·0%) |
| >1-2 MW | 40 (21·5%) | 48 (24·5%) |
| >2 MW | 39 (21·0%) | 48 (24·5%) |
| **Smoker, No. (%)** | 32 (17·0%) | 24 (12·2%) |
| **Hypertension (self-reported), No. (%)** | 114 (60·6%) | 134 (68·0%) |
| **Diabetes (self-reported), No. (%)** | 58 (30·9%) | 72 (36·6%) |
| **Receiving pharmacological treatment for depression (self-reported), No. (%)** | 12 (6·4%) | 9 (4·6%) |
| **Mean PHQ-9 scores (SD)** | 7·29 (1·35) | 6·87 (1·43) |
| **Mean GAD-7 scores (SD)** | 9·43 (4·12) | 8·89 (4·16) |
| **Mean EQ-5D-5L scores (SD)** | 0·880 (0·098) | 0·901 (0·086) |
| **Mean ICECAP-O scores (SD)** | 0·716 (0·146) | 0·729 (0·145) |
| **Mean 3-item UCLA scores (SD)** | 5·02 (1·74) | 4·89 (1·65) |

*Abbreviations:* 3-item UCLA scores: 3-item University of California, Los Angele loneliness scale; EQ-5D-5L: 5-level EuroQol health-related quality of life questionnaire; GAD-7: 7-item General Anxiety Disorder questionnaire; ICECAP-O: ICEpop CAPability measure for older people; MW: minimum wage (in 2021, the minimum wage in Brazil was BRL1110 (approximately US$213); PHQ-9: 9-item Patient Health Questionnaire; SD: standard deviation; UBS: Unidade Básica de Saúde (primary care clinic)

PHQ-9 scores range from 0 to 27, with higher scores representing more severe depressive symptomatology

GAD-7 scores ranges from 0 to 21 with higher scores representing more severe anxiety symptomatology

EQ-5D-5L scores range from -0·264 to 1, with higher scores representing higher quality of life

ICECAP-O scores range from 0 to 1, with higher scores representing greater levels of capability and general wellbeing

3-item UCLA scores range from 3 to 9, with higher scores representing more severe levels of loneliness

Supplementary Table 12: Comparison of baseline demographics and baseline measures for secondary outcomes, between trial arms, for participants followed-up at five months

|  | **Intervention group (n=175)** | **Control group (n=191)** |
| --- | --- | --- |
| **Sex, No. (%)** |  |  |
| Female | 63 (36·0%) | 68 (35·6%) |
| Male | 112 (64·0%) | 123 (64·4%) |
| **Age group, No. (%)** |  |  |
| 60-69 years | 140 (80·0%) | 155 (81·2%) |
| 70+ years | 35 (20·0%) | 36 (18·8%) |
| **Type of UBS, No. (%)** |  |  |
| Family Health Strategy | 149 (85·1%) | 160 (83·8%) |
| Mixed models | 26 (14·9%) | 31 (16·2%) |
| **Education, No. (%)** |  |  |
| None | 19 (10·9%) | 32 (16·8%) |
| 1-4 years | 35 (20·0%) | 50 (26·3%) |
| 5-8 years | 42 (24·0%) | 36 (19·0%) |
| >8 years | 79 (45·1%) | 72 (37·9%) |
| **Personal income, No. (%)** |  |  |
| Up to 1 MW | 96 (55·5%) | 103 (54·2%) |
| >1-2 MW | 37 (21·4%) | 44 (23·2%) |
| >2 MW | 40 (23·1%) | 43 (22·6%) |
| **Smoker, No. (%)** | 29 (16·6%) | 23 (12·0%) |
| **Hypertension (self-reported), No. (%)** | 107 (61·1%) | 130 (68·1%) |
| **Diabetes (self-reported), No. (%)** | 53 (30·3%) | 68 (35·6%) |
| **Receiving pharmacological treatment for depression (self-reported), No. (%)** | 12 (6·4%) | 9 (4·6%) |
| **Mean PHQ-9 scores (SD)** | 7·26 (1·35) | 6·85 (1·44) |
| **Mean GAD-7 scores (SD)** | 9·37 (4·07) | 8·82 (4·16) |
| **Mean EQ-5D-5L scores (SD)** | 0·877 (0·100) | 0·902 (0·088) |
| **Mean ICECAP-O scores (SD)** | 0·715 (0·146) | 0·734 (0·147) |
| **Mean 3-item UCLA scores (SD)** | 5·00 (1.75) | 4·90 (1·64) |

*Abbreviations:* 3-item UCLA scores: 3-item University of California, Los Angele loneliness scale; EQ-5D-5L: 5-level EuroQol health-related quality of life questionnaire; GAD-7: 7-item General Anxiety Disorder questionnaire; ICECAP-O: ICEpop CAPability measure for older people; MW: minimum wage (in 2021, the minimum wage in Brazil was BRL1110 (approximately US$213); PHQ-9: 9-item Patient Health Questionnaire; SD: standard deviation; UBS: Unidade Básica de Saúde (primary care clinic)

PHQ-9 scores range from 0 to 27, with higher scores representing more severe depressive symptomatology

GAD-7 scores ranges from 0 to 21 with higher scores representing more severe anxiety symptomatology

EQ-5D-5L scores range from -0·264 to 1, with higher scores representing higher quality of life

ICECAP-O scores range from 0 to 1, with higher scores representing greater levels of capability and general wellbeing

3-item UCLA scores range from 3 to 9, with higher scores representing more severe levels of loneliness

Sensitivity analyses checking for any potential effect of adjusting for number of elapsed days between baseline and the first follow-up assessment

An additional analysis was performed to explore the potential effect of differences in the number of days elapsed from baseline to the first follow-up between the intervention arm (median number of days (MDN) 88), inter-quartile range (IQR) 87, 90) and control arm (MDN=88, IQR, 87, 90). Estimates from models using complete cases only, that adjusted for number of days elapsed, were very similar (difference in mean PHQ-9 scores between the intervention and control arms at three months: -0·57; 95% CI: -1·69, 0·52), to the model that did not adjust for number of days elapsed (-0·64; -1·75, 0·46).

**References**

1. Lee KJ, Carlin JB. Multiple imputation for missing data: fully conditional specification versus multivariate normal imputation. *Am J Epidemiol*. Mar 01 2010;171(5):624-32. doi:10.1093/aje/kwp425

2. Sterne JA, White IR, Carlin JB, et al. Multiple imputation for missing data in epidemiological and clinical research: potential and pitfalls. *BMJ*. Jun 29 2009;338:b2393. doi:10.1136/bmj.b2393

3. Rubin DB. *Multiple Imputation for Nonresponse in Surveys*. Wiley Series in Probability and Statistics. John Wiley & Sons, Inc.; 1987:258.

4. Carpenter J, Kenward M, White I. Sensitivity analysis after multiple imputation under missing at random: a weighting approach. *Statistical methods in medical research*. June 2007;16(3):259-75. doi:10.1177/0962280206075303

5. Carpenter J, Pocock S, Lamm C. Coping with missing data in clinical trials: a model-based approach applied to asthma trials. *Stat Med*. Apr 30 2002;21(8):1043-66. doi:10.1002/sim.1065

6. Heraud-Bousquet V, Larsen C, Carpenter J, Desenclos JC, Le Strat Y. Practical considerations for sensitivity analysis after multiple imputation applied to epidemiological studies with incomplete data. *BMC medical research methodology*. 2012;12:73. doi:10.1186/1471-2288-12-73
