# Supplementary material for: A self-help mobile messaging intervention to improve subthreshold depressive symptoms among older adults in a socioeconomically deprived region of Brazil (PRODIGITAL): a pragmatic, two-arm randomised controlled trial
Source: Lancet Reg Health Am. 2024 Oct 7;39:100897. doi: 10.1016/j.lana.2024.100897 (PMC11491721; doi:10.1016/j.lana.2024.100897)
Supplement: Summary Portuguese [file mmc3.docx]

Resumo

Contexto: A depressão subclínica é um fator de risco para a depressão maior e está associada ao aumento da morbidade e mortalidade, especialmente em idosos. Estão surgindo evidências de que intervenções digitais, incluindo intervenções de autoajuda, podem reduzir os sintomas depressivos. Nosso objetivo foi avaliar a efetividade de uma intervenção realizada através do envio de mensagens pelo celular para a redução dos sintomas depressivos subclínicos entre idosos no Brasil.

Métodos: O PRODIGITAL foi um ensaio clínico randomizado controlado, simples cego e com dois grupos, conduzido em 46 Unidades Básicas de Saúde na cidade de Guarulhos, Brasil. Indivíduos com 60 anos ou mais foram contatados por telefone para uma avaliação de triagem, seguindo uma lista ordenada aleatoriamente. Aqueles que apresentaram anedonia e/ou humor deprimido (Questionário de Saúde do Paciente (PHQ)-2≥1) e que, subsequentemente, pontuaram entre 5 e 9 no PHQ-9 foram convidados a participar. O grupo intervenção recebeu a intervenção digital de autoajuda Viva Vida, consistindo em mensagens multimídia automatizadas enviadas via WhatsApp. Quarenta e oito mensagens de áudio ou visuais baseadas em psicoeducação e ativação comportamental foram enviadas automaticamente ao longo de seis semanas. O grupo controle recebeu uma única mensagem contendo informações sobre depressão. O desfecho primário foi a diferença na média dos escores do PHQ-9 entre os grupos na avaliação de seguimento de três meses. Todas as análises primárias foram realizadas de acordo com o grupo alocado e com dados imputados. O ensaio está registrado no ReBEC, RBR-6c7ghfd.

Resultados: Os participantes foram recrutados entre 8 de setembro de 2021 e 19 de agosto de 2022. Dos 454 participantes incluídos, 223 foram randomizados para o grupo intervenção e 231 para o grupo controle. A média de idade dos participantes foi 65.3 anos (DP 5.0) e 64.0% (n=292) eram mulheres. Um total de 385 (84,8%) completou a avaliação de seguimento de três meses; não foi observada diferença nos escores médios do PHQ-9 entre os grupos do estudo (diferença ajustada: -0,61; 95% IC: -1,75, 0,53; *p*=0,29).

Interpretação: Estes resultados demonstram que a intervenção digital de autoajuda Viva Vida não contribuiu para a melhoria dos sintomas depressivos subclínicos entre os idosos. Mais pesquisas são necessárias para entender porque essa intervenção não foi efetiva nessa população e para explorar como ela pode ser adaptada para atingir esse objetivo.

Financiamento: Fundação de Amparo à Pesquisa do Estado de São Paulo (FAPESP) e UK Joint Global Health Trials
